# A pediatric case of measles with gastrointestinal complications in an urban hospital setting: A clinical and public health perspective

**DOI:** 10.1016/j.idcr.2026.e02657

**Published:** 2026-06-30

**Authors:** Yahye Hassan Muse, Mukhtar Abdi Hassan, Mahdi Ali, Abdisalam Hassan Muse, Saralees Nadarajah

**Affiliations:** a Faculty of Science and Humanities, School of Postgraduate Studies and Research (SPGSR), University of Hargeisa, Hargeisa 25263, Somalia; b Mahdi Multi-specialty Hospital, Hargeisa 25263, Somalia; c Department of Mathematics, University of Manchester, Manchester M13 9PL, UK

**Keywords:** Measles, Pediatric case report, Gastrointestinal complications, Viral infection, Child health, Infectious diseases

## Abstract

**Background:**

Measles remains a significant public health concern in under-immunized populations, often leading to complications such as dehydration and secondary infections.

**Case Presentation:**

A 4.5-year-old girl from an urban area presented with fever, vomiting, diarrhea, red eyes, and poor oral intake. Clinical assessment and lab findings supported a diagnosis of measles. She was hospitalized for five days and managed with IV fluids, antibiotics, antipyretics, Vitamin A, and supportive care.

**Outcome:**

The patient showed marked improvement, with resolution of systemic symptoms and only a mild residual cough at discharge.

**Conclusion:**

This case highlights the classical presentation and effective management of measles, emphasizing the importance of early intervention and robust vaccination programs.

## Background

Measles is a highly contagious viral illness caused by the measles virus, a member of the genus *Morbillivirus* in the *Paramyxoviridae* family [1]. Despite the availability of a safe and effective vaccine, measles remains a significant cause of morbidity and mortality among young children globally, particularly in resource-limited settings or areas with suboptimal vaccination coverage [2], [3], [4].

Transmission occurs primarily through respiratory droplets produced when an infected person coughs or sneezes, which can remain infectious in the air or on surfaces for up to two hours [5], [6]. The incubation period typically averages 10–14 days from exposure to the onset of symptoms [7], [8], [9] Clinically, measles often presents with a prodromal phase lasting several days, characterized by fever, malaise, and the classic triad of cough, coryza (runny nose), and conjunctivitis (often referred to as the "three Cs") [10], [11].

Koplik spots, small white spots on the buccal mucosa opposite the molars, may appear briefly during the prodrome and are pathognomonic for measles [12]. This is followed by the appearance of a characteristic maculopapular rash, usually starting on the face and spreading downwards (cephalocaudal progression) over several days [13], [14] Complications are common and can range from mild (e.g., otitis media, diarrhea) to severe and life-threatening (e.g., pneumonia, laryngotracheobronchitis, encephalitis, and, rarely, subacute sclerosing panencephalitis years later) [15], [16].

Malnutrition, particularly Vitamin A deficiency, significantly increases the risk of severe complications and mortality. Therefore, the World Health Organization (WHO) recommends high-dose Vitamin A supplementation for all children diagnosed with measles to reduce ocular complications and overall mortality [17], [18]. Prevention through widespread vaccination with the measles-mumps-rubella (MMR) or measles-containing vaccine (MCV) is the central to measles and rubella elimination efforts [19], [20], [21]. In the local setting, measles prevention depends on routine measles-containing vaccination delivered through the national immunization programme, together with periodic supplementary immunization or catch-up activities. However, immunity gaps may remain where routine coverage is incomplete or children miss scheduled doses, creating conditions for sporadic cases or outbreaks.

## Patient information

This case report details the presentation and management of a 4.5-year-old female patient residing in an urban setting. According to the mother’s report, the child had not received any measles-containing vaccine and was therefore unvaccinated at the time of illness. The patient's mother is a 29-year-old housewife, married, and residing in the same urban environment. This demographic information provides context regarding the patient's home environment but does not inherently point to specific risk factors beyond potential exposure within the community. The patient's young age places her within the demographic most vulnerable to measles complications if unvaccinated or under vaccinated.

## Clinical presentation

The patient's illness commenced three days prior to hospital admission, initially manifesting solely as fever. Subsequently, her condition worsened with the development of vomiting and diarrhea. Concurrently, she exhibited a significant decrease in oral intake, refusing fluids and food, which contributed to her deteriorating condition. The documented history of illness confirmed the presence of fever, a skin rash (though details of its appearance and distribution were not fully specified in the provided notes, it was considered consistent with measles), persistent vomiting, ongoing diarrhea, and poor oral intake. These symptoms collectively pointed towards a systemic viral illness with significant gastrointestinal involvement and evolving dehydration, prompting the need for hospital evaluation and admission.

## Assessment and diagnosis

Upon initial assessment in the hospital, the patient presented as generally unwell. Significant findings included observable signs of dehydration, likely secondary to fever, vomiting, diarrhea, and poor fluid intake. She was febrile and exhibited bilateral conjunctival injection ("red eyes"), a hallmark sign often associated with measles infection. Based on the characteristic constellation of symptoms reported in history (fever, rash, prodromal symptoms like probable cough/coryza implied by the later treatment) and the clinical findings on examination (fever, conjunctivitis, dehydration), a clinical diagnosis of acute measles was made.

Laboratory investigations were conducted to support the diagnosis, assess severity, and evaluate potential complications. The C-Reactive Protein (CRP) level was mildly elevated at 6 mg/L, indicating an inflammatory process, consistent with a viral infection like measles but not specific. The Anti-Streptolysin O (ASO) titre was notably elevated at 715 IU/mL, which might suggest a recent streptococcal infection, although this was not the primary diagnosis and could be an incidental finding or reflect cross-reactivity. Renal function, assessed via creatinine level, was normal at 0.6 mg/dL, which was reassuring despite the clinical signs of dehydration. The Complete Blood Count (CBC) revealed a White Blood Cell (WBC) count of 4.2 × 10^9/L, which is in the low-normal range, often seen in measles due to viral suppression of hematopoiesis. The differential count showed lymphopenia (absolute lymphocyte count 0.9 ×10^9/L, 20.6%) and a relative neutrophilia (absolute granulocyte count 3.2 ×10^9/L, 76.0%), a pattern frequently observed during acute measles infection. Red blood cell parameters showed a slightly elevated RBC count (6.25 ×10^12/L) with normal Hemoglobin (12.8 g/dL) and Hematocrit (44%). However, the red cell indices (MCV 71.3 fL, MCH 20.5 pg, MCHC 28.8 g/dL) indicated a microcytic, hypochromic picture, suggestive of possible underlying iron deficiency, a common nutritional issue in this age group. The platelet count (331 ×10^9/L) and other indices like RDW, MPV, and PDW were within normal limits. The PCT value of 2.74 mL/L is noted, but the unit "mL/L" for Procalcitonin (if that is what PCT represents here) is highly unusual and difficult to interpret in this context; typically, PCT is measured in ng/mL or µg/L. Collectively, the clinical picture combined with the supportive laboratory findings strongly affirmed the diagnosis of Measles. Table 1

**Table 1:** Laboratory findings and clinical interpretation for pediatric measles case.

**Category**	**Test**	**Result**	**Normal Range**	**Interpretation**
**Inflammatory Markers**	C-Reactive Protein (CRP)	6 mg/L	< 5 mg/L	Mildly elevated (inflammation)
	ASO Titer	715 IU/mL	< 200 IU/mL	Elevated (possible past streptococcal infection)
**Renal Function**	Creatinine	0.6 mg/dL	0.3–0.7 mg/dL (children)	Normal renal function
**White Blood Cells**	WBC Count	4.2 × 10⁹/L	5–12 × 10⁹/L	Mild leukopenia
	Lymphocyte Count	0.9 × 10⁹/L	2–7 × 10⁹/L	Lymphopenia
	Granulocytes Count	3.2 × 10⁹/L	2–7 × 10⁹/L	Normal
	Lymphocyte %	20.6%	25–40%	Slightly decreased
	Granulocyte %	76.0%	45–75%	Slight increase
**Red Blood Cells**	RBC Count	6.25 × 10 ¹ ²/L	4.1–5.5 × 10 ¹ ²/L	Elevated (possible dehydration)
	Hemoglobin (Hb)	12.8 g/dL	11.5–13.5 g/dL	Normal
	Hematocrit (HCT)	44%	34–40%	Elevated (possibly due to dehydration)
	MCV	71.3 fL	75–90 fL	Low (microcytic)
	MCH	20.5 pg	25–35 pg	Low (hypochromic)
	MCHC	28.8 g/dL	30–36 g/dL	Slightly low
**Platelet Indices**	Platelet Count	331 × 10⁹/L	150–450 × 10⁹/L	Normal
	MPV	8.3 fL	7.5–11.5 fL	Normal
	PDW	16.2	9–17	Normal
	PCT	0.274 mL/L	0.2–0.5% (0.002–0.005)	Acceptable range

**Table 2:** Management plan for pediatric measles case.

**Treatment Category**	**Medication / Intervention**	**Dosage & Route**	**Frequency**	**Purpose**
**Fluid & Electrolyte**	Dextrose Normal Saline (DNS)	200 mL IV	TID	Correct dehydration, restore fluid balance
**Antiemetics**	Ondansetron	2 mg IV	BID	Control nausea and vomiting
**Antibiotic Prophylaxis**	Ceftriaxone	500 mg IV	BID	Prevent/treat secondary bacterial infection
**Gastroprotection**	Omeprazole	15 mg IV	BID	Prevent gastric irritation and protect mucosa
**Eye Care**	Tetracycline (TTC) eye ointment	Topical	BID	Treat/prevent eye infection/conjunctivitis
**Antipyretics**	Paracetamol	180 mg IV	PRN	Manage fever and discomfort
**Cough Suppression**	Toplexyl Syrup	5 mL oral	TID	Relieve persistent cough
**Micronutrient Support**	Vitamin A	200,000 IU oral	Day 1 & Day 2	Prevent complications, improve epithelial healing

## Management and outcome

The patient was admitted to the hospital and managed over a period of five working days. The treatment strategy focused on supportive care, aggressive rehydration, management of symptoms, nutritional support, and prevention/monitoring for secondary complications. Intravenous fluid therapy was initiated with Dextrose 5% in Normal Saline (DNS) at a rate of 200 mL administered three times daily (TID) to correct dehydration and maintain hydration status. Given the high risk of secondary bacterial infections (such as pneumonia or otitis media) in measles, broad-spectrum antibiotic coverage was provided with Ceftriaxone 500 mg intravenously (IV) twice daily (BID). Vomiting was managed with the anti-emetic Ondansetron 2 mg IV BID. Omeprazole 15 mg IV BID was administered, likely for gastric protection due to stress or potential gastritis from vomiting. Specific treatment for the measles-associated conjunctivitis involved the application of Tetracycline (TTC) eye ointment twice daily. Fever and discomfort were managed with Paracetamol 180 mg IV administered as needed (PRN). A persistent cough was treated symptomatically with Toplexyl syrup 5 mL TID. Crucially, adhering to WHO guidelines for measles management, the patient received high-dose Vitamin A supplementation (200,000 IU) on day 1 and day 2 of admission to reduce the risk of complications, particularly severe diarrhea and ocular damage, and decrease mortality.

Following these interventions, the patient's overall condition showed significant improvement during the hospitalization. Dehydration resolved, fever subsided, vomiting ceased, and she gradually resumed adequate oral intake. Because the child was unvaccinated, the parents were counselled that natural measles infection provides measles immunity but does not protect against rubella. Therefore, completion of age-appropriate routine immunization, including measles-rubella-containing vaccine according to national guidance, was recommended after recovery. At the time of discharge after five days, the patient was clinically stable and had no active complaints, with the exception of a residual cough. This lingering cough is a common sequela of measles, often persisting for some weeks after the acute phase, and requires monitoring but typically resolves spontaneously.

## Discussion

This case effectively demonstrates the typical presentation and standard management of acute measles in a young child, complicated by dehydration and conjunctivitis. The diagnosis was primarily clinical, supported by characteristic laboratory findings like leukopenia with lymphopenia. The management strategy appropriately prioritized fluid resuscitation, nutritional support (including the critical Vitamin A supplementation), symptomatic relief, and vigilance for secondary bacterial infections through the empirical use of antibiotics. The elevated ASO titre warrants consideration but did not alter the primary diagnosis or management of measles; it could represent a recent unrelated infection or be a non-specific finding. The microcytic, hypochromic red cell indices highlight the potential for co-existing nutritional deficiencies, like iron deficiency anemia, which is prevalent in this age group and can be exacerbated by acute illness; follow-up assessment of iron status post-recovery would be advisable.

This case underscores several important points. First, measles remains a threat, especially to unvaccinated children, and can lead to significant morbidity requiring hospitalization. Second, supportive care, particularly fluid management and nutritional support including Vitamin A, is paramount. Third, clinicians must remain vigilant for common and serious complications, justifying the use of antibiotics in many hospitalized cases. Fourth, the persistence of symptoms like cough after the acute phase is expected but requires parental education and follow-up. Finally, this case reinforces the undisputed value of measles vaccination as the most effective public health measure to prevent measles and its potentially devastating consequences. This case also illustrates the burden of a single preventable measles case on both the family and the health system. The child required hospitalization for five days, intravenous fluids, antibiotics, antipyretics, antiemetics, Vitamin A supplementation, laboratory investigations, and clinical monitoring. For the parents, this may involve transport expenses, emotional stress, and time away from work or household responsibilities. For the health system, preventable measles cases consume staff time, hospital beds, medicines, and infection prevention resources. These costs and service demands are substantially greater than the cost and effort required for timely routine immunization. Challenges in such cases include differentiating measles from other febrile rash illnesses initially and managing parental anxiety. Lessons learned include the importance of adhering to established treatment protocols (especially Vitamin A) and considering underlying health issues (like potential anemia) even during acute management.

## Conclusion

This case reflects a typical yet preventable presentation of pediatric measles with moderate complications. The outcome was favorable due to early hospitalization, prompt fluid management, and comprehensive supportive care. From a public health perspective, there is a compelling need to strengthen routine immunization programs, especially in urban settings where crowded living conditions facilitate disease transmission.

Early recognition and intervention can significantly reduce morbidity and mortality associated with measles in under-five children.

## CRediT authorship contribution statement

**Abdisalam Hassan Muse:** Writing – review & editing, Writing – original draft, Visualization, Validation, Supervision. **Mahdi Ali:** Software, Resources, Project administration. **Yahye Hassan Muse:** Data curation, Conceptualization. **Mukhtar Abdi Hassan:** Investigation. **Saralees Nadarajah:** Formal analysis, Data curation.

## Ethical approval

Not applicable. The case study does not include the patient’s name, images, or any identifiable personal information.

## Consent for publication

Written informed consent was obtained from the patient’s parents for the publication of this case report.

## Funding

Not applicable.

## Declaration of Competing Interest

The authors declare that they have no competing interests.

## Data Availability

All relevant data are included within the manuscript.
